# Behavioural and psychiatric phenotypes in female carriers of genetic mutations associated with X-linked ichthyosis

**DOI:** 10.1371/journal.pone.0212330

**Published:** 2019-02-15

**Authors:** Alice Cavenagh, Sohini Chatterjee, William Davies

**Affiliations:** 1 MRC Centre for Neuropsychiatric Genetics and Genomics and Division of Psychological Medicine and Clinical Neurosciences, School of Medicine, Cardiff University, Cardiff, United Kingdom; 2 School of Psychology, Cardiff University, Cardiff, United Kingdom; 3 Neuroscience and Mental Health Research Institute, Cardiff University, Cardiff, United Kingdom; QIMR Berghofer Medical Research Institute, AUSTRALIA

## Abstract

X-linked ichthyosis (XLI) is a rare X-linked dermatological condition arising from deficiency for the enzyme steroid sulfatase (STS). STS is normally expressed in the brain, and males with XLI exhibit personality differences from males in the general population, and are at increased risk of developmental and mood disorders. As the *STS* gene escapes X-inactivation, female carriers of XLI-associated genetic mutations have reduced STS expression/activity relative to non-carrier females, and could manifest similar behavioural phenotypes to males with XLI. Additionally, as STS activity normally increases in female tissues towards late pregnancy and into the puerperium, carrier females could theoretically present with increased rates of postpartum psychopathology. Using a worldwide online survey comprising custom-designed demographic questionnaires and multiple validated psychological questionnaires, we collected detailed self-reported information on non-postpartum and postpartum behaviour in confirmed adult (>16yrs) female carriers of genetic mutations associated with XLI (n = 94) for statistical comparison to demographically-matched previously-published normative data from female controls (seven independent studies, 98≤n≤2562), adult males with XLI (n = 58), and to newly-obtained online survey data from a general population sample of mothers from the United Kingdom and United States of America (n = 263). The pattern of results in carrier females relative to controls was remarkably similar to that previously observed in males with XLI, with evidence for increased rates of developmental and mood disorders, and elevated levels of inattention, impulsivity, autism-related traits and general psychological distress. Carrier females exhibited a significantly elevated rate of postpartum mental health conditions (notably mild depression) relative to controls which could not be accounted for by social factors. Our data confirm the psychological profile associated with XLI-associated mutations, and suggest that female carriers may be at increased risk of psychopathology, including in the postpartum period. These findings are relevant to families affected by XLI, to clinicians involved in the care of these families, and to genetic counsellors.

## Introduction

X-linked ichthyosis (XLI) is a dermatological condition characterised by large, dark brown scales occurring primarily on the extensor surfaces and trunk [1]. Most cases of XLI are caused by an X-linked genetic mutation resulting in deficiency for the enzyme steroid sulfatase (STS), which cleaves sulfate groups from a range of steroids; the skin phenotype results from accumulation of cholesterol sulfate in the stratum corneum [1]. 80–90% of XLI cases are caused by complete or partial deletion of the *STS* gene, with the remaining cases being attributed to point mutations within the gene. The typical deletion encompasses *STS* and a small number of adjacent genes (including members of the *VCX* family, *PUDP*, and *PLPNA4*) whilst larger deletions can encompass the *KAL* and *NLGN4X* genes [2]. Diagnosis of the condition is typically made on the basis of skin appearance and family history, with additional confirmatory biochemical and/or genetic analyses performed for some individuals [1]. As it arises from an X-linked mutation, XLI almost exclusively affects males: in >85% of cases of XLI, the mutation is thought to be inherited from a heterozygous carrier mother [3,4]. Prevalence rates for XLI based upon clinical samples range from 1 in 3000 to 1 in 6000 males [1], and for STS deficiency based upon prenatal screening are approximately 1 in 1500 males [5,6]. These findings suggest that, in many cases, STS deficiency does not result in clinically-identifiable disease. The inheritance data and prevalence rates above indicate that approximately 1 in 1200 females within the general population may be heterozygous carrier females, equivalent to ~30,000 individuals in the United Kingdom, and ~3.1million individuals worldwide.

*STS* is expressed in the developing [7] and adult [8] human brain, and males with XLI exhibit a personality and mental health profile distinct from that of males within the general population [2,4,9,10]; specifically, the former group exhibit higher rates of inattention, impulsivity (but not motor impulsivity), mood problems, disruptive behaviour and autism-related traits, and are more likely to be diagnosed with developmental conditions (including Attention Deficit Hyperactivity Disorder and autism) and mood disorders. Recently, a case report has identified a psychotic disorder resembling early-onset schizophrenia in a boy with XLI [11]. Importantly, these findings are unlikely to be confounded by ascertainment biases, in that high rates of psychopathology are seen in boys with XLI ascertained solely on the basis of maternal endocrine abnormalities during pregnancy [2], and a remarkably similar suite of behaviours to that reported in males with XLI is also seen *Sts*-deficient mouse models [12–16].

The *STS* gene escapes X-inactivation [17], and carrier females heterozygous for XLI-associated genetic mutations exhibit reduced STS expression, and reduced protein levels/enzyme activity [3]. Consistent with this, carrier females can show comparable, though milder, phenotypes to males with XLI [1]. The behaviour and mental health of carrier females has not been systematically assessed, although one report has described paranoid schizophrenia in two female cases with deletions encompassing *STS* [18]. In female mammals, STS expression/activity increases during late pregnancy and into the puerperium in blood and brain tissue [19], whilst carrier mothers are at increased risk of experiencing stressful obstetric complications (specifically delayed or prolonged labour due to STS absence in the fetal placenta) relative to general population controls [1]. Hence, we have previously hypothesised that deficiency for the STS enzyme might result in an increased risk of postpartum psychopathology [19], notably for postpartum psychosis-related phenotypes [20].

Here, we aimed to determine whether behavioural measures and mental health risk (including in the postpartum period) differed between female carriers of XLI-associated genetic mutations and females from the general population, and whether the pattern of differences mirrored those seen in males with XLI. Using established and effective social media-focussed recruitment methods [9,21], we undertook an online survey of confirmed female carriers of XLI-associated mutations using well-validated and detailed self-report behavioural questionnaire measures. Data related to non-postpartum behaviours were compared to previously-published demographically-matched normative sample, and adult male XLI, data. Data relevant to the postpartum period were compared to normative data obtained from a demographically-matched, newly-recruited and online-surveyed general population sample of mothers.

Our *a priori* hypotheses were that, relative to females within the general population, carrier females (similar to males with XLI) would exhibit evidence for: impaired attention and a general increase in impulsivity (but normal, or reduced, motor impulsivity), hyperactivity, increased levels of depressive and anxiety-related phenotypes, increased behavioural inflexibility and social impairment, increased rates of psychotic experiences, and a greater likelihood of having been diagnosed with one or more developmental, mood, or postpartum psychiatric disorders.

We successfully recruited, and analysed online survey data from, a comparatively-large cohort of adult female carriers of XLI-associated genetic mutations from around the world, and identified a number of behavioural differences compared to normative female samples which were largely consistent with the aforementioned hypotheses.

## Methods

### Ethics

The studies were approved by Cardiff University School of Psychology Research Ethics Committee.

### Survey structure for female carriers of XLI-associated genetic mutations

An online survey (Survey 1) was generated using Qualtrics software to be completed by confirmed adult (>16yrs) female carriers of genetic mutations associated with XLI. After an online consent form, the first part of the survey comprised demographic information including participant age, ethnic origin, country of residence, handedness, smoker status, and highest educational level attained. The second part of the survey asked about the basis on which carrier status was confirmed, and, if a genetic test was performed, the nature of the mutation. The third part of the survey asked about the participant’s sensory acuity (scored on a Likert scale from 1 = excellent to 5 = very poor for each sense), general personality type based upon the ‘Four Temperaments’ [22], and any previous diagnosis of developmental, psychiatric or neurodegenerative illness.

Participants were then asked to complete a series of five psychological questionnaires selected on the basis of previous data: i) the Adult ADHD Self-Report Scale version 1.1 (ASRS v1.1), a five-point response 18-item Likert-scale screening questionnaire assessing recent attentional (nine items) and hyperactive-impulsive (nine items) traits based on diagnostic criteria for ADHD and previously established for use in adults [23]; the presence of ≥4 symptoms (indexed by scores of ≥2 or ≥3 depending on the question) from Part A is consistent with, but not diagnostic for, a presentation of ADHD ii) the Barratt Impulsiveness Scale-11 (BIS-11), a four-point response 30-item Likert-scale questionnaire assessing aspects of impulsivity [24] for which total scores, second-order factor scores (attentional, motor and planning impulsivity) and first-order factor scores (attention, cognitive instability, motor performance, perseverance, self-control and cognitive complexity) were calculated, iii) the adult Autism Quotient (AQ), an extensively-validated four-point response 50-item Likert-scale questionnaire assessing behavioural traits associated with autism spectrum disorders [25]; total and sub-scale scores (social skill, attention-switching, attention to detail, communication and imagination) were calculated, iv) the Kessler Psychological Distress Scale (K10) [26], a five-point 10-item Likert-scale questionnaire assessing recent depression or anxiety-related traits. K10 items were scored from 1–5 (total score range 10–50) with a score ≥20 being consistent with significant psychological distress and v) the Schizotypal Personality Questionnaire, a 74-item ‘Yes/No’ screen designed to assess behavioural traits associated with schizotypal personality disorder in the general population, with items endorsed ‘Yes’ scoring one point [27]; total and sub-scale scores (items of reference, excessive social anxiety, odd beliefs or magical thinking, unusual perceptual experiences, odd or eccentric behaviour, no close friends, odd speech, constricted affect and suspiciousness) were calculated. Finally, participants completed a demographic questionnaire on their childbearing history (including any pregnancy-related medical complications or postpartum psychiatric diagnoses) and the Community Assessment of Psychic Experiences (CAPE-42) applied to the 6-week period following the birth of any of their children; the CAPE-42 is a 42-item four-point response scale designed to assess the mean frequency (1 = never to 4 = nearly always) and associated distress (1 = not distressed to 4 = very distressed) of positive, depressive and negative experiences related to psychosis within the general population [28]. The survey concluded with a short debrief describing the purpose of the study and its main hypotheses, links to relevant previous literature, and an explanation of mechanisms by which the study results would be disseminated.

### Survey structure for adult mothers from the general population

A second online survey (Survey 2) was generated using Qualtrics software to be completed by adult mothers from the general populations of United Kingdom or United States of America to provide normative behavioural data relating to the postpartum period. After completing an online consent form, participants completed a general demographic questionnaire before completing a questionnaire on their childbearing history (including any pregnancy-related medical complications or postpartum psychiatric diagnoses) and the Community Assessment of Psychic Experiences (CAPE-42) applied to the 6-week period following the birth of any of their children. The survey concluded with a short debrief similar to that for Survey 1.

### Participant recruitment and survey completion

We conservatively estimated, based upon our previous BIS-11, AQ and K10 data in adult males with XLI [9], that for two-tailed analyses with α = 0.05, a sample size of 85 female carriers would provide ~83% power to reliably detect an effect size half that observed in males. The URL for Survey 1 was advertised worldwide via ichthyosis support groups and their websites (in some cases after additional ethical and academic review), and through social media sites (including relevant Facebook pages, and Twitter). Participants completed the survey anonymously, and results were returned to the research team upon completion. Data were collected between 17^th^ November 2017 and 7^th^ June 2018. The URL for Survey 2 was advertised via the Qualtrics online participant panel [29] and respondents were reimbursed in accordance with standard Qualtrics procedures. Participants completed the survey anonymously, and results were returned to the research team upon completion. Data were collected between 8-9^th^ January 2018.

### Data analysis and statistics

Questionnaires were analysed as previously described in the literature. In the rare circumstance of participants not answering specific questions (which occurred <2.5% of the time), mean values for the group were imputed. Normality of data was assessed using Shapiro-Wilk test. Summary data are reported as percentages, mean values±standard deviation of the mean (if normally-distributed) or as median values with 95% confidence limits (if non-normally distributed); where non-normally distributed data are being directly compared to normally-distributed data, mean values±standard deviation of the mean are also presented for the former. Categorical data were analysed using Chi-Squared test with Yates’ correction or Fisher Exact Test. Testing to compare behavioural measures in the female carrier sample to the ‘average’ or mean score was done using a One Sample t-test/Wilcoxon Signed Ranks Test. Traits within the same set of individuals were compared using paired t-test or Related Samples Wilcoxon Signed Rank test. Data from female carriers of XLI-associated genetic mutations and previously-published normative control samples from seven independent studies were compared using Welch’s t-test with the degrees of freedom calculated using the Welch-Satterthwaite equation. P-values were calculated from the resultant t-statistic and degrees of freedom using online GraphPad software [30]. Data from female carriers of XLI-associated genetic mutations and adult males with XLI/general population mothers from our newly-recruited online sample were compared using categorical analyses and unpaired two-tailed t-test/Mann Whitney U-Test depending upon normality of the data. Summary descriptions of all control and comparison samples are available in **S1 Table**. P-values <0.05 were regarded as nominally-significant. *Post hoc* Principal Component Analysis (PCA) of the female carrier data for the 47 measures across ASRS, BIS-11, AQ, K10, SPQ and CAPE-42 questionnaires using SPSS 25 identified 8 distinct components explaining the majority (83%) of the total sample variance (Component 1 = 44%, Component 2 = 13%, Component 3 = 6% and Components 4–8 <5% each); thus only p-values<0.00625 (0.05/8) should be regarded as surviving conservative multiple testing correction.

### Data availability

Raw survey data are available in **S2**and **S3 Tables**.

## Results

### Recruitment and demographics of female carrier sample

A total of 104 females responded to Survey 1, 10 of whom were subsequently excluded from further analysis as their presentation was inconsistent with being a carrier for an XLI-associated mutation. The demographic features of the remaining 94 individuals, who we had high confidence were genuine carriers based upon their survey responses, are reported in **Table 1**. Although we did not genotype individuals within our female carrier sample, some participants reported their previously-defined genetic mutation; the ratio of carriers with atypical deletions (~2.5-5Mb) to carriers with typical deletions (~1.5–1.7Mb) or point mutations is consistent with previous literature [2,4].

**Table 1:** Demographic features of our sample of female carriers of genetic mutations associated with X-linked ichthyosis (XLI).

Demographic feature		Female carriers for XLI-associated mutations (n = 94)
Age (yrs)		38.5 (95%CI: 36–41)
**Country of residence**	United Kingdom	36 (38%)
	United States of America	27 (29%)
	Europe (excluding UK)	12 (13%)
	Canada	7 (7%)
	Rest of world	12 (13%)
**Handedness**	Right-handed	83 (88%)
	Left or mixed-handed	11 (12%)
**Ethnicity**	White European or Hispanic	84 (89%)
	African or Afro-Caribbean	4 (4%)
	Asian	3 (3.5%)
	Other or no response	3 (3.5%)
**Highest level of education**	No formal education	1 (1%)
	High school	23 (24.5%)
	College or University	53 (56.5%)
	Postgraduate	17 (18%)
**History of regular smoking**	Yes	30 (32%)
	No	64 (68%)
**Basis of XLI carrier status**	Family history (i.e. multiple affected male relatives) and skin appearance alone	59 (63%)
	Family history, skin appearance and genetic/hormonal test	35 (37%)
**Test result for individuals undergoing genetic analysis**	Typical deletion or mutation within *STS*	15 (43%)
	Atypical (large) deletion	3 (8.5%)
	Don’t know or no response	17 (48.5%)

### Sensory function in female carriers

Large deletions encompassing *STS* have been associated with anosmia in some cases [1], whilst *STS* is expressed in regions of the developing human tongue [7]; hence, we wanted to assess basic sensation in our female carrier cohort. Sensory function across all five domains was reported as being significantly better than average i.e. ‘3’ (p<0.001). Low numbers of individuals reported ‘very poor’ sensory function (vision: 2/94, hearing: 1/94, taste: 0/94, smell: 0/94 and touch: 0/94).

### Personality types in female carriers

Within the female carrier group (n = 93 due to no response from one participant), participants self-identified most closely with the following personality attributes: ‘optimistic and social’ (30/93, 32%), ‘short-tempered and irritable’ (29/93, 31%), ‘analytical and quiet’ (31/93, 33.5%) and ‘relaxed and peaceful’ (3/93, 3.5%). Comparing these frequencies to large-scale worldwide gender-specific data from a commercial company assessing personality traits within the general population across >2million online tests (>1million individuals) [31] indicates that within our sample there is a substantially elevated proportion of individuals self-identifying as ‘short-tempered or irritable’ (31% vs. 17.5%), and a lower proportion self-identifying as ‘analytical and quiet’ (33.5% vs. 43.5%) and ‘relaxed and peaceful’ (3.5% vs. 7%); the proportion of individuals self-identifying as ‘optimistic and social’ was equivalent within our female carrier sample and the general population sample (32% vs. 32%).

### Previous developmental, psychiatric or neurodegenerative diagnoses in the non-postpartum period in female carriers

A high proportion (39.5%) of our sample reported a previous non-postpartum diagnosis of a developmental, psychiatric or neurodegenerative condition; 29.8% reported a previous mood disorder diagnosis, 7.6% reported a previous developmental condition diagnosis, and 2.1% reported a previous mixed mood and developmental condition diagnosis (**Table 2**).

**Table 2:** Developmental and mood disorders self-reported in our sample of female carriers of genetic mutations associated with X-linked ichthyosis (XLI).

		Female carriers for XLI-associated mutations (n = 94)
**Previous diagnosis of a (non-postpartum) developmental, psychiatric or neurodegenerative condition**		37 (39.5%)
**Mood disorder**	**Anxiety disorder alone**	2 (2.1%)
	**Depression alone, or with comorbid anxiety, Obessive-Compulsive Disorder (OCD) or Post-traumatic Stress Disorder (PTSD)**	25 (26.5%)
	**Bipolar disorder alone, or with comorbid OCD**	1 (1.1%)
**Developmental conditions**	**Attention Deficit Hyperactivity Disorder (ADHD)/Attention Deficit Disorder (ADD) alone**	3 (3.3%)
	**Autism spectrum condition**	1 (1.1%)
	**Dyslexia alone**	3 (3.2%)
	**Learning disability alone**	1 (1.1%)
**Mixed mood and developmental conditions**	**Depression with comorbid ADHD**	1 (1.1%)

### Adult ADHD Self-Report Scale (ASRS) scores in female carriers

A remarkably high proportion of carrier females (45/94, 48%) met the criteria for further investigations for possible ADHD i.e. ≥4 symptoms in Part A of the screening questionnaire. When compared to a mixed-gender adult (31.1±10.3yrs) general population sample from Sweden (n = 202, with no significant difference in scores between males and females)[32], our female carrier sample scored significantly, and substantially, more highly on both the Part A screener questions (12.2±4.8 vs. 8.9±3.5, t[141] = 5.96, p<0.0001) and on the total score measure (35.3±13.5 vs. 24.6±8.7, t[129] = 7.07, p<0.0001)(Cohen’s d = 0.79 and 0.94 respectively). Within the female carrier sample, the total inattentive symptom count was significantly higher than the total hyperactive-impulsive symptom count, and the total inattention scores were significantly higher than total hyperactive-impulsive scores (both p<0.001)(**Table 3**). The three carriers of large atypical deletions demonstrated marginally elevated numbers of total symptoms (8.7±5.7) but similar total scores (36.7±11.6) to the overall sample. The total ASRS symptoms and scores across the four individuals previously diagnosed with ADHD alone, or with ADHD and comorbid depression, were 14.5±4.0 and 54.5±12.1 respectively, consistent with the questionnaire being sensitive to ADHD-relevant behaviours.

**Table 3:** Adult ADHD Self-Report Scale (ASRS) symptoms and scores in our sample of female carriers of genetic mutations associated with X-linked ichthyosis (XLI).

	Female carriers for XLI-associated mutations (n = 94)	Normative adult mixed-gender sample (n = 202, [32])	Statistical comparison
**Individuals with ≥4 symptoms in Part A**	45 (48%)	-	-
**Total symptoms**	7.5 (95%CI: 6–9)	-	-
**Inattentive symptoms**	5.0 (95%CI: 3.5–6)	-	-
**Hyperactive-impulsive symptoms**	3.0 (95%CI: 2–3)	-	-
**Total score (0–4 for all 18 items)**	35.3±13.5	24.6±8.7	t[129] = 7.07, p<0.0001
**Score (0–4 for 6 Part A items)**	12.2±4.8	8.9±3.5	t[141] = 5.96, p<0.0001
**Score (0–4 for 12 Part B items)**	23.1±94	-	-
**Score (inattention items)**	19.4±7.4	-	-
**Score (hyperactive-impulsive items)**	15 (95%CI: 13–17)	-	-

### Barratt Impulsiveness Scale-11 (BIS-11) scores in female carriers compared to female controls

All BIS-11 measures from our female carrier group (n = 92) were compared to a normative sample of 1184 adult female college student and community-recruited participants screened for Axis I psychiatric disorders and positive drug, alcohol or pregnancy tests (mean age ~21.5yrs) designed to be broadly reflective of the healthy general population of the USA [33]; total BIS-11 scores within our sample were also compared to an adult female general population sample (n = 1503, mean age ~41.5yrs) from Canada screened for severe cognitive impairments [34]. The mean total score for our female carrier sample (65.8±12.4) was towards the high end of the usual range of impulsiveness (52–71) and was significantly, and substantially, higher than that of both the USA (62.1±10.6) and Canadian (55.9±9.6) general population samples (t[101] = 2.77, p<0.01, and t[97] = 7.50, p<0.0001, Cohen’s d = 0.32 and 0.89 respectively). Individuals carrying atypical large deletions performed similarly to the overall carrier sample on the total BIS-11 score measure (66.7±7.6). The total BIS-11 score across the four individuals previously diagnosed with ADHD alone, or ADHD with comorbid depression, was extremely high (85.0±8.5) consistent with the questionnaire being sensitive to clinically-relevant impulsive traits. In terms of second order BIS-11 factors, carrier females scored more highly than USA general population controls on attentional and non-planning impulsivity, but did not differ in terms of motor impulsivity; with respect to first order factors, female carriers scored more highly than general population controls on measures of attention, cognitive instability, perseverance, and self-control, but equivalently on a measure of cognitive complexity, and lower (though not significantly so) on a specific measure of motor impulsivity (**Table 4**).

**Table 4:** Barratt Impulsiveness Scale-11 (BIS-11) scores in our sample of female carriers of genetic mutations associated with X-linked ichthyosis (XLI) and a normative general population control sample.

	Female carriers for XLI-associated mutations (n = 92)	Normative adult female sample (n = 1184, [33])	Statistical comparison
**Total score**	65.8±12.4	62.1±10.6	t[101] = 2.77, p = 0.0067
**Attentional impulsivity**	18.0±4.9	16.7±4.1	t[101] = 2.53, p = 0.01
**Motor impulsivity**	22.0 (95%CI: 20.0–23.5); 22.5±4.9	21.8±4.1	t[101] = 1.38, p = 0.17
**Non-planning impulsivity**	25.2±5.2	23.6±5.0	t[104] = 2.91, p<0.005
**Attention**	11.0 (95%CI: 10.0–12.0); 11.1±3.3	10.4±2.9	t[102] = 2.02, p = 0.05
**Cognitive instability**	7.0 (95%CI: 6.0–8.0); 6.9±2.2	6.3±1.9	t[101] = 2.54, p = 0.01
**Motor**	14.0 (95%CI: 13.0–15.5); 14.8±4.0	15.0±3.4	t[101] = -0.56, p = 0.57
**Perseverance**	8.0 (95%CI: 7.0–8.0); 7.8±2.0	6.8±1.7	t[101] = 4.58, p<0.001
**Self-control**	13.4±3.6	12.0±3.3	t[103] = 3.53, p<0.001
**Cognitive complexity**	11.9±2.6	11.6±2.6	t[105] = 0.96, p = 0.34

### Autism Quotient (AQ) scores in female carriers compared to female controls

All AQ measures from our female carrier group (n = 90) were compared to a normative sample of 98 unscreened adult female participants recruited from the UK general population (mean age ~37yrs)[25]; total AQ scores from our sample were also compared to a large adult female general population sample recruited online and screened for autism and other psychiatric disorders (n = 2562, aged 34.4±4.4yrs) [35]. The total score for our female carrier sample (21.0 (95%CI: 18.0–23.0); 21.6±8.8) was towards the high end of the usual female range for autism-related traits [25], and was significantly, and substantially, higher than that of both the smaller (15.4±5.7) and larger (17.1±7.6) normative general population samples (t[150] = 5.66, p<0.0001 and t[93] = 4.77, p<0.0001, Cohen’s d = 0.84 and 0.55 respectively). The three individuals carrying atypical large deletions scored more highly, on average, than the overall carrier sample on the total AQ score measure (30.0±11.5). The female carrier previously diagnosed with autism had a total score of 34 on the AQ, consistent with the questionnaire’s senstivity to autism-related traits. On four out of five AQ subscales, the female carrier sample scored significantly more highly than the normative control sample; however, on the ‘Attention to Detail’ subscale, there was no significant difference between the two groups’ scores (**Table 5**).

**Table 5:** Autism Quotient (AQ) scores in our sample of female carriers of genetic mutations associated with X-linked ichthyosis (XLI) and a normative general population control sample.

	Female carriers for XLI-associated mutations (n = 90)	Normative adult female sample (n = 98, [25])	Statistical comparison
**Total score**	21.0 (95%CI: 18.0–23.0); 21.6±8.8	15.4±5.7	t[150] = 5.66, p<0.0001
**Social skill**	4.0 (95%CI: 2.0–5.0); 4.0±2.9	2.3±2.2	t[164] = 4.51, p<0.0001
**Attention switching**	5.0 (95%CI: 4.5–6.0); 5.3±2.4	3.6±1.8	t[166] = 5.67, p<0.0001
**Attention to detail**	5.0 (95%CI: 4.0–6.0); 5.3±2.4	5.4±2.3	t[183] = -0.36, p = 0.72
**Communication**	3.0 (95%CI: 3.0–4.0); 3.9±2.4	2.1±1.8	t[164] = 5.70, p<0.0001
**Imagination**	2.0 (95%CI: 2.0–3.0); 3.1±2.2	1.9±1.5	t[153] = 4.16, p<0.0001

### Kessler Psychological Distress Scale (K10) scores in female carriers compared to female controls

Data from the female carrier sample (n = 91) were compared to normative data from an unscreened community-recruited Australian adult female sample comprising 882 individuals (35–44yrs) [36]. Total scores in the female carrier sample were significantly, and substantially, larger than those seen in the normative sample (23.0 (95%CI: 20.0–25.0); 23.6±8.5 vs. 15.5±8.9 respectively, t[111] = 8.63, p<0.0001), Cohen’s d = 0.93) and were in the range consistent with the presence of a mild mental disorder (20–24). Compared to the overall female carrier sample, the three female carriers of atypical deletions exhibited slightly lower than average total K10 scores (21.7±5.7). As expected, the 25 individuals with a previous diagnosis of depression alone, or depression with another comorbid psychiatric condition, had high K10 scores (29.9±8.5), consistent with a diagnosis of a moderate-severe mental disorder.

### Schizotypal Personality Questionnaire (SPQ) scores in female carriers compared to female controls

SPQ data from the female carrier sample (n = 88) were compared to normative data from an unscreened sample of adult (39.8±10.3yrs) females (n = 184) recruited from the community in Australia [37]. Female carriers exhibited significantly, and substantially, higher total SPQ scores than the normative sample (24.0 (95%CI: 20.0–28.5);25.5±15.2 vs.15.9±11.4 respectively, t[134] = 5.43, p<0.0001, Cohen’s d = 0.73). The total SPQ scores for the two female carriers with atypical deletions who completed this questionnaire were on the lower side of the average for the carrier group (16.5±6.4). On the majority of SPQ subscales, carrier females scored significantly more highly than females from the normative sample; however, on the ‘Unusual Perceptual Experiences’ measure the two groups performed equivalently, whilst the female carrier group scored nominally-significantly lower on the ‘Odd beliefs and magical thinking’ measures (**Table 6**).

**Table 6:** Schizotypal Personality Questionnaire (SPQ) scores in our sample of female carriers of genetic mutations associated with X-linked ichthyosis (XLI) and a normative general population control sample.

	Female carriers for XLI-associated mutations (n = 88)	Normative adult female sample (n = 184, [37])	Statistical comparison
**Total score**	24.0 (95%CI: 20.0–28.5); 25.5±15.2	15.9±11.4	t[135] = 5.27, p<0.0001
**Ideas of reference**	2.4±2.5	1.71±2.06	t[146] = 2.33, p = 0.02
**Excessive social anxiety**	4.0 (95%CI: 3.0–6.0); 4.4±2.8	3.04±2.39	t[150] = 3.88, p<0.0005
**Odd beliefs or magical thinking**	1.0 (95%CI: 0.0–1.0); 1.5±1.7	2.08±1.81	t[179] = -2.76, p = 0.0064
**Unusual perceptual experiences**	2.0 (95%CI: 1.0–2.0); 2.1±2.0	1.91±2.10	t[182] = 0.65, p = 0.52
**Odd or eccentric behaviour**	1.0 (95%CI: 0.5–2.0); 2.0±2.1	0.75±1.43	t[126] = 5.05, p<0.0001
**No close friends**	3.0 (95%CI: 2.0–5.5); 3.9±3.0	1.49±1.84	t[120] = 6.93, p<0.0001
**Odd speech**	4.0 (95%CI: 3.0–5.0); 3.8±2.6	2.40±2.13	t[144] = 4.32, p<0.0001
**Constricted affect**	2.0 (95%CI: 2.0–3.0); 2.7±2.3	1.05±1.28	t[114] = 6.37, p<0.0001
**Suspiciousness**	2.0 (95%CI: 2.0–3.0); 2.9±2.5	1.51±1.80	t[132] = 4.56, p<0.0001

### Relationship between psychological questionnaire scores in female carrier sample

Within the female carrier sample, all total questionnaire scores (ASRS, BIS-11, AQ, K10 and SPQ) were highly significantly correlated with one another (correlation coefficients>0.48, p<0.001) apart from BIS-11 and AQ total scores which were moderately significantly correlated (Spearman’s rho = 0.22, p = 0.04).

### Comparison of demographics and psychological questionnaire scores in female carrier sample and previous sample of adult males with XLI

We compared the demographics and total ASRS, BIS-11, AQ and K10 scores from our female carrier sample to a previously-ascertained adult male sample with XLI [9](**Table 7**). The two samples were well-matched for most demographic variables, although our female carrier sample was significantly more highly-educated. On average, total questionnaire scores were consistently lower in the female carrier than in the male XLI group, but they were only significantly lower in the case of the AQ and K10 measures (and only nominally so for the AQ) (**Table 7**).

**Table 7:** Demographics and total psychological questionnaire scores in our sample of female carriers of genetic mutations associated with X-linked ichthyosis (XLI) and adult males with XLI.

		Female carriers for XLI-associated mutations (n = 91–94)	Males with XLI (n = 44–58, [9])	Statistical comparison
**Demographic measure**	**Age (yrs)**	38.5 (95%CI: 36–41)	39.5 (95%CI: 33–47)	U = 2716.5, p = 0.97
	**Country of residence** **(UK/USA/Other)**	36 (38%)/27(29%)/31(33%)	23(40%)/19(32%)/16(28%)	χ^2^[2] = 0.55, p = 0.76
	**Handedness** **(Right/Left or Mixed)**	83(88%)/11(12%)	49(84%)/9(16%)	χ^2^[1] = 0.18, p = 0.67
	**Ethnicity** **(White European or Hispanic/Other)**	84(89%)/10(11%)	46(79%)/12(21%)	χ^2^[1] = 2.17, p = 0.14
	**Highest level of education** **(No formal or high school/college or University/postgraduate)**	24(26%)/53(56%)/17(18%)	24(41%)/32(55%)/2(4%)	χ^2^[2] = 9.01, p = 0.01
**Psychological questionnaire measure**	**Total ASRS score**	35.3±13.5	36.7±11.6	t[141] = -0.60, p = 0.55
	**Total BIS-11 score**	65.8±12.4	68.8±14.1	t[137] = -1.32, p = 0.19
	**Total AQ score**	21.0 (95%CI: 18.0–23.0); 21.6±8.8	23.9±5.9	U = 1604.5, p = 0.05
	**Total K10 score**	23.0 (95%CI: 20.0–25.0); 23.6±8.5	28.4±9.5	U = 1393.0, p<0.005

### Comparison of demographics of female carrier mothers and general population sample of UK and US mothers

83 of our female carrier sample had given birth to at least one child, and we recruited 263 mothers from the general UK and USA populations as a control sample. The control sample, on average, were marginally younger at survey completion than the female carrier sample, had their first child at a slightly younger age, and had slightly fewer children overall; the two samples did not differ significantly on any other demographic measure we assessed (**Table 8**).

**Table 8:** Demographics of our sample of female carriers of genetic mutations associated with X-linked ichthyosis (XLI) and our adult female general population sample of mothers.

Demographic variable	Female carrier mothers for XLI-associated mutations (n = 83)	General UK and USA population sample of mothers (n = 263)	Statistical comparison
**Age (yrs)**	39.0 (95%CI: 37.0–42.0)	37.0 (95%CI: 35.0–38.0)	U = 8287.5, p = 0.001
**Age at first child (yrs)**	27.4±5.4	25.0 (95%CI: 24.0–26.0); 25.5±5.7	U = 6650.5, p = 0.005
**Number of children**	2.0 (95%CI: 2.0–2.0)	2.0 (95%CI: 2.0–2.0)	U = 9138.0, p = 0.04
**Handedness** **(Right/Left or Mixed)**	75(90%)/8(10%)	233(89%)/30(11%)	χ^2^[1] = 0.06, p = 0.81
**Ethnicity** **(White European or Hispanic/Other)**	75(90%)/8(10%)	237(90%)/26(10%)	χ^2^[1] = 0.02, p = 0.89
**Highest level of education** **(No formal or high school/college or University/postgraduate)**	22(27%)/45(54%)/16(19%)	75(29%)/154(59%)/33(12%) (1 no response)	χ^2^[2] = 2.31, p = 0.32
**Smoking status** **(Regular smoker/Non-smoker)**	30(36%)/53(63%)	123(47%)/140(53%)	χ^2^[1] = 2.47, p = 0.12

### Pregnancy and postpartum-related medical complications and mental health conditions in female carriers compared to general population mothers

As expected, female carriers of mutations associated with XLI exhibited a significantly, and substantially (>threefold), higher rate of delayed or prolonged labour relative to females from the general population; the two groups did not differ with respect to any of the other pregnancy or childbirth-related medical conditions we examined (**Table 9**).

**Table 9:** Pregnancy and childbirth-related medical complications in our sample of female carriers of genetic mutations associated with X-linked ichthyosis (XLI) and our adult female general population sample of mothers; note that one individual could have experienced multiple complications.

		Female carrier mothers for XLI-associated mutations (n = 83)	General UK and USA population sample of mothers (n = 263)	Statistical comparison
**Medical complications during pregnancy or childbirth**	**Delayed or prolonged labour**	52(63%)	50(19%)	χ^2^[1] = 55.7, p<0.0001
	**Premature labour**	17(20%)	53(20%)	χ^2^[1] = 0.01, p = 0.92
	**Loss of pregnancy or miscarriage**	17(20%)	63(24%)	χ^2^[1] = 0.25, p = 0.62
	**Pre-eclampsia or high blood pressure**	14(17%)	40(15%)	χ^2^[1] = 0.04, p = 0.84
	**Gestational diabetes**	12(14%)	20(8%)	χ^2^[1] = 2.76, p = 0.10
	**Thyroid problems**	5(6%)	14(5%)	χ^2^[1] = 0.0, p = 1.00

Female carriers exhibited a significantly elevated (~1.5fold) burden of all aggregated postpartum mental health conditions, and ‘depressive’ postpartum mental health conditions, relative to general population controls (**Table 10**). Importantly, this finding was unlikely to be due to the former group being exposed to more adverse external social circumstances during pregnancy and postnatally; in fact there was a trend for the opposite to be the case (**Table 10**). For specific postpartum conditions, only the prevalence of ‘baby blues’ (mild depression not requiring further medical treatment) was significantly higher in the female carrier group (**Table 10**). Both females carrying large atypical deletions reported experiencing the ‘baby blues’ following one or more pregnancies. We tested whether there was any relationship between stressful childbirth i.e. delayed or prolonged labour and an increased rate of postpartum mental health conditions in our carrier sample: 26 female carriers experienced both delayed/prolonged labour and a postpartum mental health condition, 26 delayed/prolonged labour but no postpartum mental health condition, 12 no delayed/prolonged labour but a postpartum mental health condition, and 19 no delayed/prolonged labour and no postpartum mental health condition (χ^2^[1] = 0.59, p = 0.44). We also tested the postpartum-specificity of our findings by assessing whether carrier females who reported a postpartum mental health condition (n = 38) scored more highly than those who didn’t (n = 45) on the K10 ‘baseline’ (non-postpartum) measure of depressive and anxiety-related traits; whilst the former group scored more highly, on average, on the K10 (25.5±9.0 vs. 22 (95%CI: 19.0–24.0); 22.4±8.0), this was not significant (U = 672.5, p = 0.10). Female carriers who reported a postpartum mental health condition were not currently older, did not have their first child later, and did not have more children, than female carriers who did not report a postpartum mental health condition; age: 41.7±9.4yrs vs. 41.0 (95%CI: 35.0–46.0); 42.6±11.8yrs (U = 838.0, p = 0.88), age at first child: 27.9±5.0yrs vs. 26.9±5.7yrs (t[70] = 0.77, p = 0.44), and number of children: 2.0 (95%CI: 2.0–2.0) vs. 2.0 (95%CI: 2.0–2.0)(U = 855.0, p = 1.00) respectively.

**Table 10:** Postpartum mental health conditions in our sample of female carriers of genetic mutations associated with X-linked ichthyosis (XLI) and our adult female general population sample of mothers; note that one individual could have experienced multiple conditions.

	Female carrier mothers for XLI-associated mutations (n = 83)	General UK and USA population sample of mothers (n = 263)	Statistical comparison
**Presence of any postpartum mental health condition**	38(46%)	83(32%)	χ^2^[1] = 5.00, p = 0.03
**Postpartum depressive condition (‘baby blues’ or postpartum depression)**	34(41%)	70(27%)	χ^2^[1] = 5.51, p = 0.02
**Obvious external social factors contributing to postpartum mental health condition (Yes/No)**	15(39%)/17(45%) (6 no response (16%))	52(63%)/30(36%) (1 no response (1%))	χ^2^[1] = 1.96, p = 0.16
**‘Baby blues’ or mild depression not requiring further medical treatment**	23(28%)	39(15%)	χ^2^[1] = 6.27, p = 0.01
**Postpartum depression requiring further medical treatment**	16(19%)	38(14%)	χ^2^[1] = 0.78, p = 0.38
**Postpartum psychosis**	1(1%)	3(1%)	p = 1.00
**Postpartum anxiety**	6(7%)	19(7%)	χ^2^[1] = 0.06, p = 0.81
**Postpartum post-traumatic stress disorder (PTSD)**	6(7%)	10(4%)	p = 0.23
**Postpartum obsessive-compulsive disorder (OCD)**	2(2%)	6(2%)	p = 1.00
**Postpartum-onset bipolar disorder**	0(0%)	6(2%)	p = 0.34

### Community Assessment of Psychic Experiences (CAPE-42) scores related to the postpartum period in female carrier and general population mothers

We attempted to obtain a quantitative measure of positive, depressive and negative experiences related to psychosis in the postpartum period using the CAPE-42 [28] in our female carrier and general population samples. Female carriers reported significantly less frequent positive dimension psychosis-related experiences than females from the general population, but these experiences, when they occurred, caused equivalent amounts of distress in both groups (**Table 11**). Whilst the average frequency of depressive psychosis-related experiences (and the distress caused by these) was greater in the female carrier group, the two groups did not differ significantly; nor was there any significant group diffference with respect to the frequency of, or distress caused by, negative dimension psychosis-related experiences (**Table 11**). The two females carrying large atypical deletions scored similarly to the overall carrier sample (≤2 on all measures). Within the female carrier group, those reporting postpartum mental health conditions (n = 37) scored significantly higher on depressive (frequency and distress) and negative (frequency and distress) dimension experiences compared to those not reporting such conditions (n = 44): 2.45±0.69 vs. 2.09±0.63 (t[77] = 3.43, p = 0.001), 2.63±0.72 vs. 2.25±0.62 (t[77] = 3.54, p = 0.001), 2.14 (95%CI:1.79–2.57) vs. 1.79 (95%CI:1.57–2.14) (U = 504.5, p = 0.003), and 2.09±0.57 vs. 1.87±0.42 (t[77] = 2.34, p = 0.02) respectively; these two groups did not differ with respect to positive dimension experience frequency (1.20 (95%CI:1.10–1.25) vs. 1.25 (95%CI:1.20–1.30)(U = 721.5, p = 0.38)) or associated distress (2.00 (95%CI:1.77–2.11 vs. 1.60 (95%CI:1.25–2.00)(U = 366.5, p = 0.11)). Hence, the CAPE-42, as applied to the postpartum period, does appear to be sensitive to mental health symptoms.

**Table 11:** Community Assessment of Psychic Experiences-42 (CAPE-42) scores in our sample of female carriers of genetic mutations associated with X-linked ichthyosis (XLI) and our adult female general population sample of mothers.

	Female carrier mothers for XLI-associated mutations (n = 81)	General UK and USA population sample of mothers (n = 263)	Statistical comparison
**Positive dimension (frequency)**	1.25 (95%CI:1.18–1.30)	1.28 (95%CI:1.25–1.35)	U = 8088.5, p = 0.001
**Positive dimension (distress)**	2.00 (95%CI:1.61–2.00)	2.00 (95%CI: 2.00–2.00)	U = 6159.5, p = 0.11
**Depressive dimension (frequency)**	2.25 (95%CI:2.00–2.38)	2.13 (95%CI:2.06–2.25)	U = 10488.0, p = 0.83
**Depressive dimension (distress)**	2.47±0.68	2.31 (95%CI:2.17–2.40); 2.35±0.77	U = 9643.0, p = 0.55
**Negative dimension (frequency)**	2.00 (95%CI:1.71–2.14)	1.96 (95%CI:1.86–2.07)	U = 10422.5, p = 0.77
**Negative dimension (distress)**	2.00±0.50	2.00 (95%CI:1.97–2.10); 2.02±0.68	U = 8780.0, p = 0.13

## Discussion

The pattern of non-postpartum psychiatric and behavioural traits demonstrated across multiple converging measures in our well-powered sample of carrier females bears remarkable similarities to that exhibited by adult males affected by XLI [9] i.e. a moderately increased prevalence of developmental/mood diagnoses (4.3% ADHD vs. ~2.5% in general adult female population [38], 1.1% autism vs. ~0.1% in general adult female population [39] and 27.6% depression vs. ~26.7% lifetime prevalence in adult female USA general population aged 35-49yrs [40]), together with generally elevated inattentive, irritable, impulsive, and depression, autism and anxiety-related traits relative to gender-matched control subjects, but with equivalent, or reduced, levels of motor impulsivity and ‘attention to detail’. The relatively high rate of developmental conditions within our female carrier sample is in line with data from the DECIPHER database [41] in which related phenotypes (e.g. developmental delay, language impairments, intellectual disability, autism and inattention) are frequently seen in female carriers with comparatively small genetic deletions (<2Mb) encompassing *STS* (**S4 Table**). The consistency of findings across female carriers and males with XLI (and, additionally, with rodent model work), indicates that our previously-reported observations regarding the neurobehavioural features associated with STS deficiency are robust and generalisable. Here, we further showed that female carriers generally exhibit higher levels of schizotypal personality-related traits than age and gender-matched controls, although with respect to ‘positive’ psychotic experience-related measures the former group scored lower or equivalently; schizotypal personality-related traits were not assayed in males with XLI. The observation that individuals carrying atypical deletions were largely similar to individuals carrying smaller mutations, apart from on the AQ where they scored slightly higher, is consistent with the idea that a gene absent in atypical but not atypical deletions (most likely *NLGN4X*) is relatively specifically involved in autism-related behavioural phenotypes [2]. Our observation that multiple questionnaire scores were strongly correlated with one another suggests one or more possibilities regarding the effects of STS deficiency: a) it has pleiotropic effects across multiple brain regions/psychological processes, the magnitude of which can vary between individuals, b) it impacts predominantly upon one core cognitive/psychological process common to multiple behavioural phenotypes e.g. attention, or c) it impacts upon one or more psychological processes in early development and subsequent adult behaviours are a response to the effects of this e.g. early high levels of autism-related traits may feasibly result in a later expression of depression and anxiety-related traits through a variety of mechanisms, including increased victimisation [42]. Future work involving detailed longitudinal characterisation of neuropsychological differences in carrier females and males with XLI should help to resolve which of these mechanisms is most plausible. It is also important to recognise that psychological traits in female carriers may conceivably be affected by both biological factors (STS deficiency and its physiological consequences) and environmental/social factors e.g. the stress of raising a child, or children, with a long-term medical condition (XLI).

In males with XLI, the STS enzyme is generally completely absent, whereas in carrier females it is expected to be haploinsufficient; hence, after allowing for normal gender differences in the expression of some psychological traits, we expected carrier females to demonstrate milder phenotypes. Whilst autism and depression/anxiety-related traits were significantly lower in carrier females than males with XLI, the two groups did not differ significantly with respect to ADHD-related traits or impulsivity (although average values were slightly lower in carrier females); these latter findings suggest that any true difference between carrier females and males with XLI (who are known to be at significantly elevated risk of ADHD) on ADHD-relevant measures is likely to be relatively small.

With respect to medical conditions associated with pregnancy and childbirth, we specifically observed the expected, and marked, elevation in prolonged/delayed labour in female carriers relative to females from the general population; this ‘control’ result provides confirmatory evidence that self-identified ‘carriers’ for XLI-associated genetic mutations are likely to be true carriers, and suggests that participants provided largely-accurate retrospective medical histories.

We successfully managed to recruit and phenotype a closely demographically-matched general population sample of mothers to compare with our female carrier sample. Importantly, self- and retrospectively-reported rates of postpartum psychiatric diagnoses in this normative sample were highly-consistent with previous epidemiological evidence from females within the general population [43] i.e. 14% vs. ~13% for postpartum depression, and 7% vs. ~10% for postpartum anxiety. Consistent with one of our original hypotheses, we identified a significantly, and substantially (~1.5-fold), higher burden of aggregated postpartum psychiatric conditions in our female carrier sample relative to the control sample; this group difference was primarily driven by a higher prevalence of depressive conditions (particularly in the mild category) and could not be readily explained by differential adverse social circumstances between the groups. We previously proposed that STS deficiency may predispose to postpartum psychosis [20]. Here, we found no evidence that carrier status confers very large increases in postpartum psychosis risk, but given both the low prevalence and the likely complex and polygenic nature of this condition, and our limited sample size, the possibility that STS deficiency moderately increases risk cannot be completely discounted. However, using the CAPE-42 measure applied to the postpartum period, we found no evidence for an increased rate of psychosis-related experiences across the female carrier group relative to the normative sample; indeed, consistent with the SPQ finding, the frequency of positive dimension experiences was actually lower in the carrier group.

The increased rate of depressive postpartum disorders in our female carrier sample could potentially have been due to the fact that this group was slightly older than the normative sample, had their children later (increased maternal age is a risk factor for postpartum mood conditions [44]), and had more children (hence, more opportunity for developing postpartum mental health problems); however, the size of the between-group differences on these demographic variables was small, and we found no evidence that female carriers reporting, or not reporting, postpartum mental health conditions differed with respect to any of these demographic measures. Our data suggest no clear relationship between delayed/prolonged labour and later postpartum psychiatric distress in female carriers, although given the relatively low power of our sample for this analysis a subtle relationship between these variables cannot be definitively ruled out; this would be interesting to explore further given comparatively high rates of caesarian births in female carriers (due to prolonged labour) and a putative relationship between caesarian birth and postpartum stress [45]. As female carriers with postpartum psychiatric diagnoses did not differ significantly from those with no postpartum psychiatric diagnosis on the K10 measure of recent depression and anxiety-related traits, we tentatively speculate that STS deficiency in late pregnancy and the postpartum period may exacerbate any non-postpartum psychiatric symptoms at these timepoints.

Our study has a number of limitations, notably: a) a lack of objective clinical and genetic confirmation, b) the possibility of response bias whereby more severely affected individuals are more motivated to participate in the study, and c) a reliance upon previously-published normative data and self, and retrospectively-reported, data. Additionally, as referred to above, the findings are simply descriptive and cannot shed light upon the mechanistic processes via which STS deficiency might ultimately impact upon physiology. We argue that these limitations do not significantly impact upon our main conclusions for the following reasons: a) participants were only included in the analyses if their carrier status was highly likely, and, consistent with this, our carrier sample exhibited expected physiological and behavioural features (i.e. high rates of delayed/prolonged labour and significant phenotypic overlap with males with XLI), b) there is no strong *a priori* reason to suspect response bias in that female carrier status has traditionally been considered to have little, or no, effect on phenotype, and c) carriers were explicitly compared to large control cohorts representative of the general population which were demographically very well (but not perfectly) matched, and for which data had been obtained in the same manner (i.e. by retrospective self-report on exactly the same measures).

### Conclusions

The current data, together with previous work, help to confirm a psychological and psychiatric profile for individuals with STS deficiency; future basic science in males with XLI, in female carriers, and in rodent models will aim to identify and characterise the biological (neural and endocrine) substrates which mediate the behavioural and psychiatric phenotypes described above; these analyses could provide clinically-relevant information on possible new therapeutic targets, and insights of more general relevance to the pathophysiology of idiopathic developmental and mood disorders. Previous work assaying *STS* gene expression in developing human brain, and on the neurochemical and genetic sequelae of STS loss of function in rodents, has provided initial clues as to neural substrates requiring more focussed investigation: specifically, these studies have implicated corticostriatothalamic, hippocampal, and cerebellar (dys)function, changes in the cholinergic and serotonergic systems, and a suite of gene expression changes [7,13,14,19,46,47]. We have previously argued that acute inhibition of STS in the postpartum period in mice may elicit effects on maternal behaviour via increased brain expression of CCN protein family members [48]; the recent finding that CTGF (also known as CCN2) is a prodepressant [49] suggests that elevated expression of this protein could partially explain the excess of postpartum depressive phenotypes in our carrier female sample.

We hope that the present information will help to continue to raise awareness of behavioural/psychiatric features associated with XLI (and carrier status for associated genetic mutations) amongst: a) families affected by the condition, b) relevant medical professionals (General Practitioners, dermatologists, psychologists/psychiatrists and obstetricians), c) patient support charities and groups, and d) genetic counsellors, such that affected individuals have the best possible access to advice and multidisciplinary medical care should it be required. Finally, the current findings, and planned future work, may be useful for understanding the basis of neurobehavioural/cognitive abnormalities including elevated autism, ADHD and anxiety risk in females with Turner syndrome (X-monosomy) in which STS expression is haploinsufficient [50–52].

## Supporting information

S1 Table Description of the various control samples compared to our newly-recruited female carrier sample. (DOCX)

S2 Table Demographic, psychiatric and behavioural data from female carrier sample. (XLSX)

S3 Table Demographic, psychiatric and behavioural data from control sample of mothers from the USA and UK. (XLSX)

S4 Table Developmental and behavioural phenotypes in female carriers of small (<2Mb) deletions spanning *STS* from the DECIPHER database. (DOCX)
