# Supplementary material for: Behavioural and psychiatric phenotypes in female carriers of genetic mutations associated with X-linked ichthyosis
Source: PLoS One. 2019 Feb 15;14(2):e0212330. doi: 10.1371/journal.pone.0212330 (PMC6377116; doi:10.1371/journal.pone.0212330)
Supplement: S1 Table — (DOCX) [file pone.0212330.s001.docx]

**S1 Table. Description of the various control samples compared to our newly-recruited female carrier sample**

| **Psychological measure** | **Origin of control sample** | **Size of control sample** | **Age of control sample** |
| --- | --- | --- | --- |
| Overall personality type | Females from worldwide general population (respondents to commerical company [1]) | Number of females not specified (>1million males and females combined) | Not specified |
| Adult ADHD Self-Report Scale (ASRS) | Mixed-gender sample from general population of Sweden [2] | n=202 | 31.1±10.3yrs |
| Barratt Impulsiveness Scale (BIS-11); total and subscale scores | Female college student and community-recruited participants from United States of America screened for Axis I psychiatric disorders and positive drug, alcohol or pregnancy tests [3] | n=1184 | Mean age ~21.5yrs |
| Barratt Impulsiveness Scale (BIS-11); total score | Female general population sample from Canada screened for severe cognitive impairments [4] | n=1503 | Mean age ~41.5yrs |
| Autism Quotient (AQ); total and subscale scores | Unscreened female participants recruited from the United Kingdom general population [5] | n=98 | Mean age ~37yrs |
| Autism Quotient (AQ); total score | Female general population sample recruited online and screened for autism and other psychiatric disorders [6] | n=2562 | 34.4±4.4yrs |
| Kessler Psychological Distress Scale (K10) | Unscreened sample of females recruited from the community in Australia [7] | n=882 | Age range 35-44yrs |
| Schizotypal Personality Questionnaire (SPQ) | Unscreened sample of females recruited from the community in Australia [8] | n=184 | 39.8±10.3yrs |
| Total ASRS, BIS-11, AQ and K10 scores | Males with X-linked ichthyosis recruited worldwide via charities and social media [9] | 44<n<58 | Median age 39.5yrs |
| Postpartum mental health condition frequency and Community Assessment of Psychic Experiences (CAPE42) total and subscale scores | Mothers recruited from general population of United Kingdom and United States of America (new sample for present study) | n=263 | Median age 37.0yrs |

1. Unknown author. Frequency of personality types [cited 11 July 2018]. Available: <http://www.mypersonality.info/personality-types/population-gender/>
2. Edebol H, Helldin L and Norlander T (2013) Measuring adult Attention Deficit Hyperactivity Disorder using the Quantified Behavior Test Plus. Psych J 2: 48-62.
3. Stanford MS, Matthias CW, Dougherty DM, Lake SL, Anderson NE and Patton JH (2009) Fifty years of the Barratt Impulsiveness Scale: An update and review. Personality and Individual Differences 47: 385-395.
4. Reyes Allyon A (2012) Impulsivity in men and women: A general population study in the Southwest of Montreal. Department of Psychiatry. McGill University.
5. Baron-Cohen S, Wheelwright S, Skinner R, Martin J and Clubley E (2001) The autism-spectrum quotient (AQ): evidence from Asperger syndrome/high-functioning autism, males and females, scientists and mathematicians. J Autism Dev Disord 31: 5-17.
6. Baron-Cohen S, Cassidy S, Auyeung B, Allison C, Achoukhi M, Robertson S, et al. (2014) Attenuation of typical sex differences in 800 adults with autism vs. 3,900 controls. PLoS One 9: e102251.
7. Slade T, Grove R and Burgess P (2011) Kessler Psychological Distress Scale: normative data from the 2007 Australian National Survey of Mental Health and Wellbeing. Aust N Z J Psychiatry 45: 308-316.
8. Badcock JC and Dragovic M (2006) Schizotypal personality in mature adults. Personality and Individual Differences 40: 77-85.
9. Chatterjee S, Humby T and Davies W (2016) Behavioural and Psychiatric Phenotypes in Men and Boys with X-Linked Ichthyosis: Evidence from a Worldwide Online Survey. PLoS One 11: e0164417.
