# Supplementary material for: Behavioural and psychiatric phenotypes in female carriers of genetic mutations associated with X-linked ichthyosis
Source: PLoS One. 2019 Feb 15;14(2):e0212330. doi: 10.1371/journal.pone.0212330 (PMC6377116; doi:10.1371/journal.pone.0212330)
Supplement: S4 Table — (DOCX) [file pone.0212330.s004.docx]

**S4 Table.** **Developmental and behavioural phenotypes in female carriers of small (<2Mb) deletions spanning *STS* from the DECIPHER database.** Open access females in DECIPHER v9.20 database (accessed 12.02.18) with small (<2Mb) deletions encompassing STS (X:7219456-7354810) and their associated phenotypes. This study makes use of data generated by the DECIPHER community [1]. A full list of centres who contributed to the generation of the data is available from [http://decipher.sanger.ac.uk](http://decipher.sanger.ac.uk/) and via email from [decipher@sanger.ac.uk](mailto:decipher@sanger.ac.uk). Funding for the project was provided by the Wellcome Trust.

| **DECIPHER ID** | **Deletion size and location** | **Inheritance pattern of mutation** | **Pathogenicity; contribution** | **Associated phenotype** |
| --- | --- | --- | --- | --- |
| 1585 | 1.73Mb: 6495281-8221971 | De novo constitutive | Unknown | Abnormality of the hair. Abnormality of the palmar creases. Deep plantar creases. High palate. Hypertelorism. Hypoplasia of the corpus callosum. Intellectual disability. Long face. Pointed chin. Posteriorly rotated ears. |
| 2369 | 1.72Mb: 6441957-8159608 | Inherited from parent with similar phenotype to child | Unknown | Intellectual disability |
| 248980 | 1.48Mb: 6551155-8032120 | De novo constitutive | Unknown | No phenotype reported |
| 250554 | 1.64Mb: 6488721-8131810 | Inherited from normal parent | Unknown | No phenotype reported |
| 253514 | 1.57Mb: 6457403-8032120 | Inherited from parent with similar phenotype to child | Unknown | No phenotype reported |
| 253515 | 1.57Mb: 6457403-8032120 | Inherited from normal parent | Unknown | No phenotype reported |
| 253516 | 1.57Mb: 6457403-8032120 | Unknown | Unknown | No phenotype reported |
| 255229 | 1.57Mb: 6457403-8032061 | Inherited from normal parent | Unknown | No phenotype reported |
| 256000 | 1.57Mb: 6457403-8032120 | Inherited from normal parent | Unknown | No phenotype reported |
| 256001 | 1.47Mb: 6457403-7923723 | Inherited from normal parent | Unknown | No phenotype reported |
| 256671 | 1.68Mb: 6463086-8144213 | Inherited from normal parent | Unknown | Abnormality of the small intestine. Generalized tonic seizures. |
| 257305 | 1.51Mb: 6562512-8075294 | De novo constitutive | Unknown | Intellectual disability. Strabismus. |
| 257574 | 1.12Mb: 6628293-7744161 | Unknown | Unknown | No phenotype reported |
| 261073 | 1.04Mb: 6705268-7744191 | Unknown | Unknown | No phenotype reported |
| 261565 | 1.56Mb: 6552712-8115153 | Inherited from normal parent | Unknown | No phenotype reported |
| 262103 | 1.56Mb: 6552712-8118153 | Unknown | Unknown | No phenotype reported |
| 265107 | 1.68Mb: 6455151-8135644 | Unknown | Unknown | No phenotype reported |
| 267306 | 1.32Mb: 6755142-8078155 | Unknown | Unknown | No phenotype reported |
| 270383 | 1.68Mb: 6453048-8133172 | De novo constitutive | Unknown | Autistic behaviour. Moderate global developmental delay. Postnatal microcephaly. |
| 270900 | 1.65Mb: 6451805-8097481 | Unknown | Unknown | No phenotype reported |
| 276070 | 1.07Mb: 6896184-7964736 | Inherited from normal parent | Unknown | Feeding difficulties in infancy. Global developmental delay. Intrauterine growth restriction. |
| 277869 | 1.56Mb: 6552712-8115153 | Inherited from parent with unknown phenotype | Unknown | No phenotype reported |
| 280020 | 1.54Mb: 6552712-8097511 | De novo constitutive | Definitely pathogenic; partial | Moderate intellectual disability |
| 283062 | 1.56Mb: 6552712-8115153 | Maternally inherited constitutive in mother | Unknown | Aggressive behaviour. Global developmental delay. Short attention span. Short stature. |
| 284832 | 1.68Mb: 6451701-8131781 | Unknown | Unknown | Cognitive impairment. Delayed speech and language development. |
| 294651 | 1.56Mb: 6552712-8115153 | Paternally inherited; constitutive in father | Likely pathogenic | No phenotype reported |
| 294916 | 1.56Mb: 6552712-8115153 | Unknown | Likely pathogenic | Behavioural abnormality. Cognitive impairment. Seizures. |
| 294958 | 1.61Mb: 6488521-8097652 | Unknown | Unknown | Hemolytic anaemia. Vertebral segmentation defect. |
| 300652 | 1.56Mb: 6552712-8115153 | Maternally inherited; constitutive in mother | Uncertain | Abnormality of movement. Absent speech. Intellectual disability. |
| 332794 | 1.13Mb: 6726925-7861330 | Maternally inherited; constitutive in mother | Uncertain; uncertain | No phenotype reported |
| 338884 | 1.64Mb: 6489877-8131810 | De novo constitutive | Definitely pathogenic; full | Autism. Delayed speech and language development. |
| 339534 | 1.66Mb: 6454368-8115153 | Paternally inherited; constitutive in father | Uncertain | Abnormal facial shape. Delayed speech and language development. Global developmental delay. |
| 351459 | 1.61Mb: 6488721-8097511 | De novo constitutive | Uncertain; uncertain | Increased nuchal translucency |
| 359354 | 1.61Mb: 6488721-8097511 | Unknown | Uncertain | Abnormal facial shape |

[1] Firth HV, Richards SM, Bevan AP, Clayton S, Corpas M, Rajan D, et al. (2009) DECIPHER: Database of Chromosomal Imbalance and Phenotype in Humans Using Ensembl Resources. Am J Hum Genet 84: 524-533.
